# Protocol for the generation and immunostaining of thick murine lung tissue slices

**DOI:** 10.1016/j.xpro.2025.104034

**Published:** 2025-08-18

**Authors:** Ali Khadim, Mahtab Shahriari Felordi, Hawlader Sabbir Hasan, Georgios Kiliaris, Mahsa Zabihi, Hannah Hofmann, Elie El Agha

**Affiliations:** 1 Department of Medicine V, Internal Medicine, Infectious Diseases and Infection Control, Universities of Giessen and Marburg Lung Center (UGMLC), German Center for Lung Research (DZL), Justus Liebig University Giessen, 35392 Giessen, Germany; 2 Cardio-Pulmonary Institute (CPI), 35392 Giessen, Germany; 3 Institute for Lung Health (ILH), 35392 Giessen, Germany

**Keywords:** Developmental biology, Microscopy, Molecular Biology

## Abstract

Precision-cut lung slices (PCLSs) largely preserve the native structural and cellular complexity of the lung, enabling detailed spatial analysis of resident cell populations. Here, we present a protocol for generating PCLSs from mouse lungs for subsequent immunofluorescent staining to detect protein expression. We describe steps for preparing animals and instruments and generating PCLSs. We then detail procedures for immunofluorescence for ACTA2 while preserving the endogenous fluorescent reporter in PCLSs and co-staining ACTA2 and MKI-67 with the endogenous reporter in PCLSs.

For complete details on the use and execution of this protocol, please refer to Khadim et al.^1^

## Highlights

• Guidance on murine lung perfusion, inflation, and cutting into thick sections
• Steps for immunofluorescence while preserving the endogenous reporter
• Instructions for antigen retrieval and recovery of the endogenous reporter
• Procedures for troubleshooting weak or unspecific immunofluorescent signals

## Abstract (editor highlights)

Publisher’s note: Undertaking any experimental protocol requires adherence to local institutional guidelines for laboratory safety and ethics.

## Graphical abstract

**Figure:**
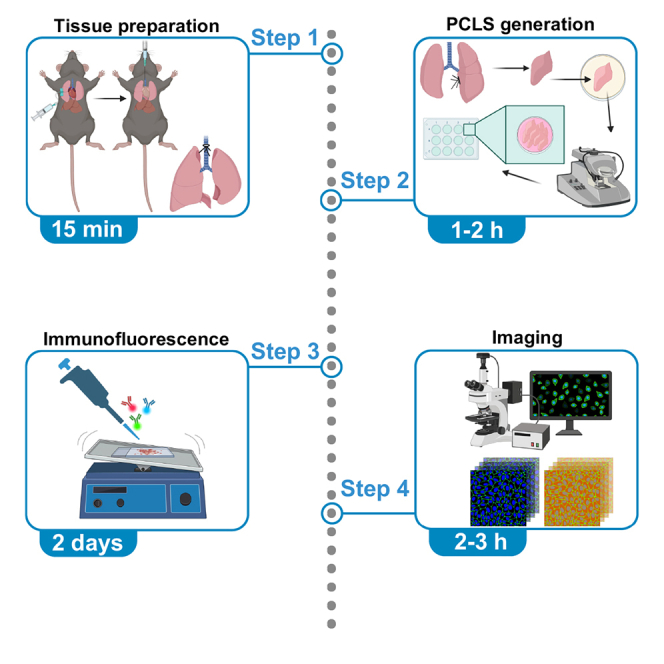

## Before you begin

The development of PCLS has transformed experimental lung research by enabling investigations in viable, structurally intact tissue that retains native cellular interactions and responses.^2^ Before starting this protocol, carefully consider the specific research objectives and the parameters that may affect slice viability and staining quality such as mouse age, strain, and health status. All reagents, including freshly prepared low-melting agarose and cold buffers, should be available, and slicing equipment must be clean and calibrated in advance. Slices generated by this protocol can be immediately used for fixed or live imaging, short-term cultures, or stimulation assays, making the method adaptable for diverse applications, from studying acute injury to testing therapeutics.

Animal welfare and scientific rigor are foundational to this process. All procedures involving live animals must strictly adhere to local and national ethical regulations. Ensure protocols for anesthesia, perfusion, and euthanasia are both effective and humane, minimizing procedural artifacts. Aim to maximize tissue yield from each animal; one mouse lung can yield dozens of slices, thus reduce overall animal use and support the principles of 3R (Replacement, Reduction and Refinement). Planning these steps carefully ensures reproducibility and the highest ethical standards while enabling meaningful translational research.

### Institutional permissions

All animal procedures were conducted in compliance with institutional guidelines and approved by the Animal Ethics Committee of Justus-Liebig University Giessen and local authorities (Regierungspraesidium Giessen; approval number G57/2021).

### Tamoxifen administration and animal handling

Mice were maintained under specific pathogen-free (SPF) conditions with ad libitum access to food and water. To achieve targeted genetic labeling, we utilized our recently developed *Fgf10*^*Cre-ERT2*^ mice,^1^^,^^3^ while *tdTomato*^*flox*^ mice were obtained from the Jackson Laboratory. *Fgf10*^*Cre-ERT2*^; *tdTomato*^*flox*^ mice were heterozygous for the *Fgf10*^*CreERT2*^ allele and homozygous for the floxed tdTomato reporter. In these mice, tamoxifen-induced, Cre-mediated recombination excised the STOP cassette at the *Rosa26* locus, resulting in permanent tdTomato expression in *Fgf10*-expressing cells and their progeny. Recombination was induced in 8–16-week-old adult *Fgf10*^*Cre-ERT2*^; *tdTomato*^*flox*^ mice by intraperitoneal (i.p.) injection of tamoxifen (0.1 mg/g body weight) every other day over three total injections, two weeks prior to tissue collection. Tamoxifen was dissolved at 20 mg/mL in corn oil. Both male and female mice were used in all experiments.

### Reagent preparation

**Timing: 10 min**

1. Preparation of ultra-low melting point (ULMP) agarose:
  a. To prepare a 2% (w/v) ULMP agarose solution, accurately weigh 600 mg of ULMP agarose and add it to 30 mL of sterile phosphate-buffered saline (PBS) in a 50 mL Falcon tube.
  b. Secure the tube upright using a plastic holding stand. Cover the tube opening with plastic wrap and puncture a small hole to allow steam to escape.
  c. Heat the tube in a microwave at 100 W until bubbles begin to form.
  d. Carefully remove the tube and gently swirl to resuspend any undissolved agarose.
    **CRITICAL:** Exercise caution when handling the tube, as the solution may be overheated and can foam or boil over upon swirling.
  e. Reheat in short intervals until the solution reaches a gentle boil and all agarose is fully dissolved.
  f. Once clear, carefully remove the tube and gently swirl to ensure uniform mixing.
  g. Transfer the tube to a 37°C incubator to maintain the agarose in liquid form until use.

## Key resources table

**Table:** 

REAGENT or RESOURCE	SOURCE	IDENTIFIER
**Antibodies**		

Mouse monoclonal anti-actin, α-smooth muscle – FITC (1:200)	Sigma	Cat# F3777-.2ML; RRID:AB_476977
**Alternative:** α-actin antibody (1A4) Alexa Fluor 488 (1:200)	Santa Cruz Biotechnology	Cat# sc-32251 AF488
Rabbit polyclonal anti-KI67 antibody (1:100)	Thermo Fisher Scientific	Cat# PA5-19462; RRID: AB_10981523
**Alternative:** Rabbit anti-KI67 (1:150)	Abcam	Cat# ab15580
Goat anti-rabbit IgG (H + L) highly cross-adsorbed secondary antibody, Alexa Fluor 647 (1:1,000)	Thermo Fisher Scientific	Cat# A-21245; RRID: AB_2535813

**Chemicals, peptides, and recombinant proteins**		

DAPI (4′,6-diamidino-2-phenylindole, dihydrochloride)	Thermo Fisher Scientific	Cat# D1306
Bovine serum albumin (IgG-free, protease-free)	Jackson ImmunoResearch	Cat# 001-000-162
Tamoxifen powder	Sigma	Cat# T5648-1G
Corn oil	Sigma	Cat# C8267-500ML
Amphotericin B	Sigma	Cat# A2942-100ML
UltraPure LMP agarose	Invitrogen	Cat# 16520-100
PBS (phosphate-buffered saline) (10x)	Thermo Fisher Scientific	Cat# 70011044
HBSS (Hank’s balanced salt solution)	Thermo Fisher Scientific	Cat# 14175095
Helizyme	B. Braun	Cat# 18767
Universal agarose, peqGOLD	Avantor Science Central	Cat# 9012-36-6
Sodium azide ReagentPlus, ≥99.5%	Sigma	Cat# S2002-5G
Paraformaldehyde (PFA) 4%	Merck	Cat# 104005
ROTIHistofix	Carl Roth	Cat# P087.3
Amphotericin B solution	Sigma	Cat# A2942-100ML
HEPES N-2-hydroxyethylpiperazine-N-2-ethanesulfonic acid	Gibco	Cat# 15630056
Sodium citrate dihydrate	Sigma	Cat# W302600-1KG-K
DMEM/F-12	Thermo Fisher Scientific	Cat# 11320033
Penicillin-streptomycin solution	Gibco	Cat# 15140-148
Fetal bovine serum (FBS)	Gibco	Cat# A5670701

**Experimental models: Organisms/strains**		

Mouse: Fgf10^Cre-ERT2^	El Agha Lab	Chu et al.^3^, Khadim et al.^1^
Mouse: B6; 129S6-Gt(ROSA)26Sor^tm9(CAG-tdTomato)Hze^/J	Jackson Laboratory	Cat# 007905; RRID:IMSR_JAX:007905

**Software and algorithms**		

Leica Application Suite X	https://www.leica-microsystems.com/products/microscope-software/p/leica-las-x-ls/	RRID:SCR_013673

**Other**		

Leica VT1200 vibrating blade microtome	Leica Biosystems	Cat# 14048142066
General purpose laboratory microwave	N/A	N/A
IKA Vibrax VXR	IKA	Cat# 0002819000
μ-Slide 4-well glass bottom	ibidi	Cat# 80427
12-well plate	Sarstedt	Cat# 4022421
10 mL syringe	B. Braun	Cat# 4606108V
Blade	Wilkinson Sword	Cat# 7005115E
Disposable scalpel	Feather	Cat# 19030893
Superglue	Pattex	Cat# 2822331

## Materials and equipment

**Table:** Complete DMEM/F12 medium

Reagent	Final concentration	Amount
DMEM/F12	N/A	86.5 mL
HEPES (1 M stock)	15 mM	1.5 mL
Penicillin-Streptomycin (100X stock)	1% (v/v)	1 mL
Fetal Bovine Serum (FBS)	10% (v/v)	10 mL
Amphotericin B (250 μg/mL)	1% (v/v)	1 mL
Total	N/A	100 mL

Store at 4°C after filter-sterilization for no more than one week.

**Table:** Blocking/permeabilization buffer for immunofluorescence

Reagent	Final concentration	Amount
Bovine serum albumin (BSA)	5% (w/v)	50 mg
Triton X-100	0.3% (v/v)	3 μL
PBS	N/A	997 μL
Total	N/A	1 mL

Blocking/permeabilization buffer should be prepared fresh or stored at 4°C for up to one week.

**Table:** Antigen retrieval buffer

Reagent	Final concentration	Amount
Sodium Citrate dihydrate (mw: 294.10 g/mol)	87.4 mM	12.85 g
Citric Acid (mw: 192.12 g/mol)	12.6 mM	1.21 g
Triton X-100	1% (v/v)	5 mL
Tween-20	1% (v/v)	5 mL
Distilled water	NA	∼500 mL total

The antigen retrieval buffer should be freshly prepared prior to each use.

### Storage conditions

DAPI aliquots can be stored at −20°C for up to six months to maintain stability and effectiveness. Ensure aliquots are protected from light during storage. Antibodies can be stored at 4°C or −20°C according to the manufacturer’s recommendations to preserve integrity. Avoid repeated freeze-thaw cycles to prevent degradation. Tamoxifen (dissolved in corn oil) should be stored in dark conditions at −20°C. Protect from light to prevent photodegradation of the compound. Buffers must be brought to 18°C–28°C before use and handled using sterile techniques to prevent contamination. Following fixation, PCLS can be stored in PBS containing 0.05% sodium azide at 4°C for up to two years.

**CRITICAL:** Reagents such as tamoxifen, DAPI, and PFA are hazardous and must be handled with extreme caution. Follow proper safety protocols and use appropriate personal protective equipment (PPE) when working with these chemicals.

## Step-by-step method details

### Preparation of animals and instruments

**Timing: ∼15 min per animal**

This section describes the procedures for mouse anesthesia, lung perfusion, and agarose instillation to prepare lung tissues for sectioning. These steps ensure optimal preservation of the native pulmonary structure for subsequent analysis.

***Note:*** Clean all instruments thoroughly with an enzymatic detergent (For e.g., Helizyme).

1. Anesthetize the mouse deeply using isoflurane or an approved chemical anesthetic until complete loss of pedal reflex is confirmed.
2. Place the animal in a supine position on a dissection board and secure limbs with tape or pins.
3. Disinfect the thoracic area with 70% ethanol.
4. Perform a midline skin incision from abdomen to neck and open the thoracic cavity by cutting through the rib cage to expose the heart and lungs.
5. Insert a 25G needle into the right ventricle for transcardiac perfusion using 1x HBSS (pre-warmed to 37°C, approximately 10–20 mL) and simultaneously snip the left atrium to allow blood to drain. (Alternatively, PBS may be used for perfusion and washing steps if HBSS is not available.)
6. Ensure the lungs blanch completely, confirming effective perfusion (Figure 1A).

**Figure 1:**
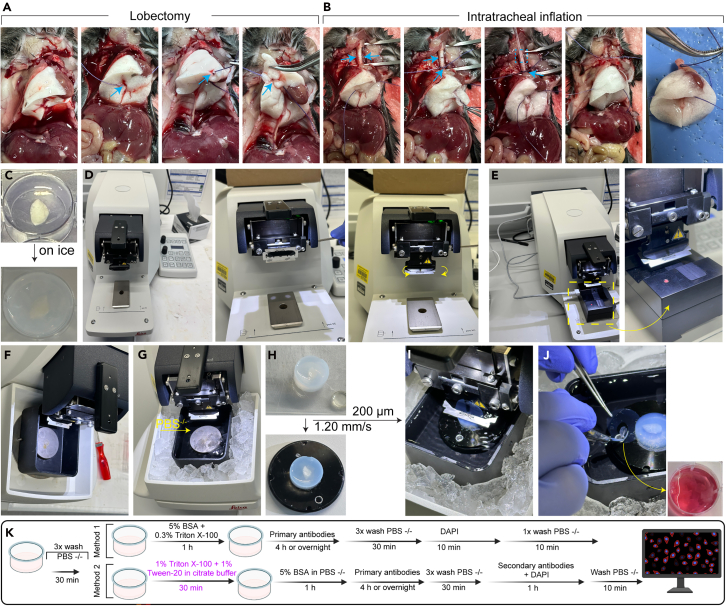
Schematic workflow for tissue isolation and generation of precision-cut lung slices (PCLS) (A) Surgical dissection of the animal, including ligation of accessory lobe and lobectomy. (B) Intratracheal instillation of low-melting-point (ULMP) agarose to ensure uniform inflation of the lung tissue. (C) Embedding of the inflated lung tissue in 5% agarose for structural support. (D–F) Setup and calibration of the vibratome and blade positioning. (G) Preparation of the specimen holder tray. (H) Mounting of the agarose-embedded lung tissue cassette onto the vibratome holding plate. (I and J) Sectioning of the embedded lung tissue using vibrating blades to generate PCLS, followed by collection of the slices for downstream applications. (K) Schematic representation of the immunofluorescent staining workflow for PCLS. BSA: Bovine serum albumin; DAPI: 4′,6-diamidino-2-phenylindole dihydrochloride; PBS: Phosphate-buffered saline.
7. Cannulate the trachea using a blunted 20G catheter, securing it with a suture thread.
8. Slowly instill 2% low-melting-point agarose (warmed to 37°C) into the lungs via the tracheal cannula (∼1–2 mL) until the lung lobes are inflated but not overdistended.

**CRITICAL:** Clamp or tie the trachea to prevent backflow and allow 10–15 min to solidify the agarose within the lung parenchyma (Figure 1B). To maintain sterility and accelerate solidification, place the filled lung in a 50 mL Falcon tube containing cold sterile medium or buffer during this step.

9. After solidification, carefully dissect out the lung and rinse in cold sterile HBSS.

***Note:*** Timing may vary depending on the number of animals processed simultaneously.

### Precision-cut lung slice generation

**Timing: 1 h**

Precision-cut lung slices (PCLS) provide a robust ex vivo platform for studying lung architecture, cell-specific responses, and molecular signaling in a near-native tissue environment. Given the delicate and highly compliant nature of lung tissue, embedding the lungs in agarose is critical—not only does it provide mechanical support, but it also prevents the collapse of alveolar structures during vibratome slicing. This step preserves the intricate lung architecture and enables the reproducible generation of uniform slices ideal for immunostaining, imaging, and culture-based assays.

10. Further embed the lungs in 5% agarose in a 6-well-cell culture plate for additional stability (Figure 1C).

**CRITICAL:** Maintain the lung in its correct anatomical orientation throughout the embedding process (For e.g., the cardiac notch of the left lobe should face downward) to avoid any rotational misalignment. If necessary, use forceps to gently position the sample slightly into the agarose.

11. After the agarose has fully solidified, carefully excise the agarose-embedded lung from the well using a feather fine scalpel, ensuring minimal disturbance to the tissue and surrounding agarose.
  a. Set vibratome parameters: Slice thickness: 200–300 μm; speed: 1.20 mm/s; amplitude: high.
    **CRITICAL:** To obtain uniform PCLS, calibrate the vibratome after placing the sectioning blade (Figures 1D–1F).
  b. Fill the vibratome chamber with cold PBS and chill the bath to 4°C with ice (Figure 1G). Alternatively, if the slices will be maintained for culturing, cold sterile culture medium (e.g., DMEM) can be used in place of PBS to support tissue viability during sectioning.
  c. Mount the embedded lung tissue onto a vibratome stage using superglue or tissue clamps (Figure 1H).
  d. Section the tissue to obtain PCLS and transfer them immediately to cold PBS containing 0.05% sodium azide for storage at 4°C (for long-term storage) or to a 12-well plate with appropriate buffer for immediate staining.
    ***Note:*** If the PCLS will be used for live imaging, collect them in sterile complete DMEM/F12 medium and incubate them at 37°C, 5% CO_2_ for at least 1 h for tissue recovery (Figure 1J).
12. Fix the slices in 4% PFA (≈ 1–2 mL per slice in a 12-well plate) at 18°C–28°C for 30 min.
13. Wash PCLS three times in 1x PBS (≈ 2 mL per slice), 10 min each, on a gentle rocking platform to remove fixative residues.

**CRITICAL:** Ensure thorough removal of PFA to reduce background and avoid interference with antibody binding.

### Immunofluorescence

This part outlines fixation, permeabilization, and immunolabeling steps to detect proteins of interest within lung slices. Careful optimization of staining protocols is essential for specific and robust signal detection.

### Part 1: Immunofluorescence for ACTA2 while preserving the endogenous fluorescent reporter in PCLS (no antigen retrieval)

**Timing: 8 h or 2 days**

**CRITICAL:** Throughout the staining procedures, PCLS remain free-floating in the wells of a 12-well plate and are transferred between solutions as needed. After staining and washing steps are complete, slices are carefully transferred to a μ-Slide 4 Well Glass Bottom chamber for imaging. At no point are the slices fixed to microscope slides; all manipulations prior to imaging are performed with the slices floating in solution.

14. Incubate slices in blocking and permeabilization buffer for 1 h at 18°C–28°C with gentle rocking, using 1–2 mL per slice in a 12-well plate (or enough buffer to fully cover each slice).

**CRITICAL:** Triton X-100 allows penetration of antibodies into the tissue; overexposure may weaken the fluorescent reporter (i.e., tdTomato in this case), so do not exceed 0.3%.

15. Prepare anti-ACTA2 antibody solution in blocking buffer at 1:200 dilution. Do not exceed the recommended antibody concentration; excessive binding can increase background and reduce specificity.
  a. Incubate slices 8 h at 4°C with gentle rocking or static incubation in a humidified chamber at 18°C–28°C for 4 h.
16. Wash PCLS three times in 1x PBS for 10 min each to remove unbound primary antibodies.
17. Incubate with DAPI diluted 1:5000 in 1x PBS for 10 min, then wash once in 1x PBS.
18. Proceed to imaging or store at 4°C in 0.05% sodium azide dissolved in sterile 1x PBS.

### Part 2: Co-staining of ACTA2 and MKI-67 with the endogenous reporter in PCLS (with antigen retrieval)

The following steps can be followed to visualize nuclear proteins such as MKI-67 by using antigen retrieval to unmask epitopes after step 14.

19. Perform antigen retrieval.
  a. Preheat sodium citrate buffer to 80°C–85°C. Allow it to cool to 37°C in a water bath and add 1% Triton X-100 and 1% Tween-20.
  b. Submerge PCLS in Luke-warm buffer for 30 min, maintaining the buffer at 37°C throughout the incubation to ensure optimal antigen retrieval conditions.

**CRITICAL:** Avoid adding PCLS to overheating buffer (above 60°C) as it can alter tissue morphology as well as fluorescent proteins.

20. Wash PCLS two times in 1x PBS, 5 min each, on a gentle rocking platform to remove the retrieval buffer.
21. Incubate slices with 1x PBS containing 5% BSA for 1 h at 18°C–28°C with gentle rocking.
22. Prepare anti-ACTA2 and anti-MKI-67 antibody solution in blocking buffer at 1:200 dilution.
  a. Incubate slices 8 h at 4°C with gentle rocking or static incubation in a humidified chamber at 18°C–28°C for 4 h.
23. Wash PCLS three times in 1x PBS for 10 min each to remove unbound primary antibodies.
24. Prepare anti-rabbit Alexa Fluor 647-conjugated secondary antibodies in blocking buffer at 1:1000 dilution.
  a. Incubate for 2 h at 18°C–28°C, protected from light.
  b. Wash PCLS three times in 1x PBS for 10 min each to remove unbound secondary antibodies.
25. Incubate with DAPI diluted at 1:5000 in 1x PBS for 10 min, then wash once in 1x PBS.
26. Proceed to imaging or store at 4°C in 0.05% sodium azide dissolved in sterile 1x PBS.

## Expected outcomes

**Figure 2:**
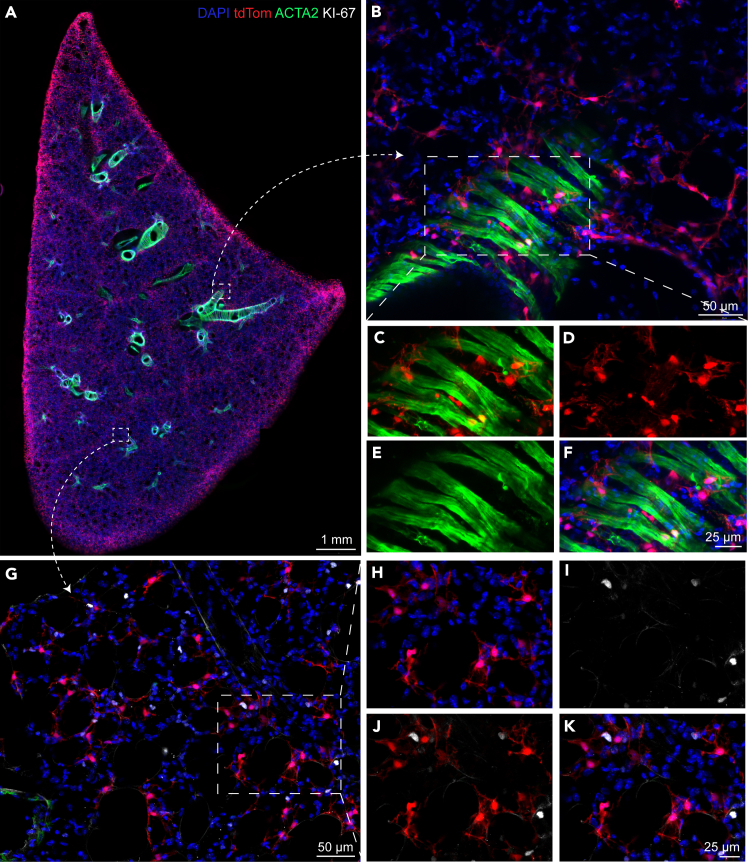
Precision-cut lung slices (PCLS) retain native 3D tissue architecture and spatial protein localization (A) Representative immunofluorescent image of PCLS stained with the indicated antibodies, demonstrating preservation of lung microanatomy. Scale bar: 1 mm. (B and G) Higher magnification views of the boxed regions in (A), highlighting specific structural features. Scale bar: 50 μm. (C–F and H–K) Further magnified views of individual fluorescence channels from the boxed areas in (B) and (G), respectively, showing detailed localization of target proteins. Scale bar: 25 μm. ACTA2: Actin Alpha 2, Smooth Muscle; DAPI: 4′,6-diamidino-2-phenylindole dihydrochloride; Ki-67: Proliferation-related Ki-67 antigen; tdTom: tdTomato fluorescent protein (reporter). Protein staining visualized in this figure was performed using protocol part 2.

This protocol enables the generation of high-quality PCLS that preserve the native architecture of lung tissues, including alveolar structures and conducting airways. When performed correctly, researchers can expect to obtain uniform slices approximately 200-300 μm in thickness, which remain intact throughout the immunostaining workflow. Alternatively, they can be kept viable for culturing purposes including live imaging. The use of ULMP agarose ensures structural stability during vibratome sectioning, while maintaining sufficient porosity for antibody penetration. Using this detailed protocol, it is expected to perform co-localization analysis of FITC-conjugated ACTA2 with lineage-labeled cells (Figures 2A–2F) in addition to the MKI-67 signal (For e.g., Alexa Fluor 647-conjugated) that marks all active phases of the cell cycle, indicating proliferation (Figures 2G–2K). This approach also allows visualizing well-preserved three-dimensional tissue structures suitable for confocal microscopy and volumetric analysis. Provided that wash steps and fixative removal are thoroughly performed, low-background fluorescence can be achieved.

When maintained in sterile culture medium post-sectioning, PCLS can be used for live-imaging applications, short-term culture assays, and drug treatment/screening experiments, making this protocol broadly applicable for ex vivo studies of lung biology, injury, fibrosis, and regeneration.

## Limitations

While PCLS offer a physiologically relevant ex vivo platform, several factors may limit the reproducibility and reliability of this important preclinical research tool. Proper inflation with ULMP agarose is critical; under- or over-inflation can result in distorted or fragile slices. Moreover, slice thickness and tissue handling significantly affect viability and antibody penetration. Dense or fibrotic regions may hinder uniform staining, especially for nuclear markers like MKI-67. Additionally, this protocol is not well suited for in situ hybridization, which is considerably more challenging in thick PCLS compared to paraffin-embedded or cryosectioned tissues due to limited probe diffusion and increased background signal.

## Troubleshooting

### Problem 1

Incomplete blanching of the lungs during perfusion.

### Potential solution

This problem leads to residual blood and compromises downstream imaging or staining quality (related to Step 5 of tissue preparation). Incomplete perfusion can result from suboptimal needle placement in the right ventricle or insufficient perfusion pressure, leading to poor clearance of blood from the pulmonary vasculature. To overcome this, ensure proper placement of the needle into the right ventricle and avoid piercing the septum. Observe the heart carefully for backflow or swelling, which may indicate incorrect positioning. Confirm adequate perfusion pressure by elevating the syringe or using a peristaltic pump if available. Snipping the left atrium early in the procedure facilitates rapid blood drainage and avoids perfusion backpressure.

***Note:*** While cold PBS is commonly used to minimize enzymatic activity and preserve tissue morphology, please be aware that using cold PBS during perfusion may lead to uneven agarose diffusion due to premature gelling or temperature-induced differences in tissue compliance. To avoid this, perfusion can also be performed with PBS at 18°C–28°C, especially if immediate agarose inflation is planned. Maintain consistent perfusion volume (10–20 mL) to ensure thorough clearance of blood.

### Problem 2

Overinflation of lungs during agarose instillation.

### Potential solution

This issue can cause tissue rupture or distortion of the alveolar architecture (related to Step 8 of tissue preparation). Overdistended lung lobes may exhibit non-physiological architecture, reducing the reliability of imaging-based assays. Therefore, slowly instill agarose using minimal pressure allowing passive inflation until lobes visibly expand but do not tense. Monitor the inflation process under a dissecting microscope to avoid overfilling. Reduce the agarose volume (e.g., closer to 150 μL) for smaller or more fragile specimens such as young mice or fibrotic lungs. Additionally, ensure that the ULMP agarose has cooled down to approximately 37°C–40°C before intratracheal instillation. Instilling agarose at 18°C–28°C should be avoided, as it may result in uneven distribution or clogging due to rapid gelation.

### Problem 3

Poor slice integrity or inconsistent thickness during vibratome sectioning (related to Step 10 of PCLS generation).

### Potential solution

Irregular or fragmented slices compromise experimental reproducibility and make imaging or quantification unreliable. Thus, when embedding the lobes in 5% agarose to maximize stability during cutting, ensure that agarose has fully solidified, and the lung is properly supported before slicing. Calibrate the vibratome blade carefully before each session to ensure uniform sectioning. Keep the vibratome bath at 4°C with ice and use fresh, high-quality razor blades to minimize drag and vibration.

### Problem 4

Weak or inconsistent antibody signal during immunofluorescence (related to immunofluorescence Step 14 and Step 19).

### Potential solution

Inadequate signal may result from poor antibody penetration, over-fixation, or inappropriate antibody concentration. Verify that Triton X-100 is present at the recommended concentration (0.3%) in the permeabilization buffer to facilitate antibody entry. Optimize primary antibody concentration within the recommended range and avoid exceeding dilutions that may increase background noise. For thick slices, consider extending antibody incubation times (For e.g., 24 h) to enhance penetration.

### Problem 5

Unspecific antibody binding or tissue autofluorescence during immunofluorescence.

### Potential solution

Unspecific antibody binding and tissue autofluorescence can confound interpretation of immunofluorescence results. To distinguish true signal from background, always include appropriate controls in your experimental design:

• **No-primary antibody control:** Incubate slices with only the secondary antibody to assess background staining from the secondary antibody itself.
• **Isotype control:** Use an isotype-matched control antibody at the same concentration as the primary antibody to evaluate non-specific binding.
• **Unstained control:** Include slices that have not been exposed to any antibodies to measure intrinsic tissue autofluorescence.

These controls help determine the specificity of antibody staining and guide adjustment of imaging acquisition parameters, such as exposure time and laser intensity, to minimize background and optimize signal-to-noise ratio.

To specifically address autofluorescence, consider the following solutions:

**Quenching treatments:** Apply chemical quenching agents such as Sudan Black B, sodium borohydride, or Eriochrome Black T after fixation and before antibody incubation to reduce tissue autofluorescence, especially in the green and red channels.

**Optimized imaging:** Adjust imaging acquisition parameters (e.g., laser intensity, exposure time) and, if available, use spectral unmixing or fluorescence lifetime imaging to separate autofluorescence from specific signals.

**Fixative choice:** When possible, use paraformaldehyde instead of formalin, as it may result in lower tissue autofluorescence.

## Resource availability

### Lead contact

Further information and requests for resources and reagents should be directed to and will be fulfilled by the lead contact, Elie El Agha (elie.el-agha@innere.med.uni-giessen.de).

### Technical contact

Technical questions on executing this protocol should be directed to and will be answered by the technical contact, Ali Khadim (ali.khadim@innere.med.uni-giessen.de).

### Materials availability

This study did not generate new unique reagents.

### Data and code availability

This study did not generate/analyze datasets and codes.

## Acknowledgments

The authors would like to thank Thomas Sontag and Ingrid Henneke for their help with the animal protocols. E.E.A. was funded by the German Research Foundation (DFG; EL 931/5-1, EL 931/4-1, EL 931/2-2 [KFO309 284237345], and SFB 1213 project ID 268555672), Institute for Lung Health (ILH), Excellence Cluster Cardio-Pulmonary Institute (CPI) (EXC2026 390649896), and German Center for Lung Research (DZL).

## Author contributions

Conceptualization, A.K. and E.E.A.; investigation, A.K., M.S.F., H.S.H., and E.E.A.; methodology, A.K., M.S.F., H.S.H., G.K., M.Z., H.H., and E.E.A.; resources, E.E.A.; writing – original draft, A.K., M.S.F., and E.E.A.; revision, A.K. and E.E.A.; funding acquisition, E.E.A.; supervision, E.E.A.

## Declaration of interests

The authors declare no competing interests.
